# Factors associated with health-related quality of life in a large national sample of patients receiving opioid substitution treatment in Germany: A cross-sectional study

**DOI:** 10.1186/s13011-018-0187-9

**Published:** 2019-01-03

**Authors:** Lisa Strada, Christiane Sybille Schmidt, Moritz Rosenkranz, Uwe Verthein, Norbert Scherbaum, Jens Reimer, Bernd Schulte

**Affiliations:** 1 Centre for Interdisciplinary Addiction Research, University Medical Centre Hamburg-Eppendorf, Martinistraße 52, 20246 Hamburg, Germany; 2 LVR-Hospital Essen, Department of Addictive Behaviour and Addiction Medicine, Medical Faculty, University of Duisburg-Essen, Essen, Germany; 3 Gesundheit Nord, Kurfürstenallee 130, 28211 Bremen, Germany

**Keywords:** Health-related quality of life, Opioid dependence, Treatment, Epidemiology, Physical health, Mental health

## Abstract

**Background:**

Knowledge of health-related quality of life (HRQOL) of patients receiving opioid substitution treatment (OST) is limited and fragmented. The present study examines the HRQOL of a large national sample of OST patients in Germany and sociodemographic and clinical correlates.

**Methods:**

Cross-sectional data on the HRQOL of 2176 OST patients was compared with German general population norms. Patients were recruited from 63 OST practices across Germany. To identify correlates of HRQOL, as measured with the SF-12, we performed bi- and multivariate analyses with sociodemographic and clinical variables, including patient- and clinician-reported outcomes on physical and mental health.

**Results:**

Patients’ HRQOL was significantly poorer than in the general population, especially their mental HRQOL. Factors associated with lower physical HRQOL were older age, longer duration of opioid dependence, hepatitis C virus infection, and HIV infection. Benzodiazepine use was associated with lower mental HRQOL, and amphetamine use with higher physical HRQOL, compared to non-use of these substances. For both mental and physical HRQOL, the factor with the strongest positive association was employment and the factors with the strongest negative associations were physical and mental health symptom severity, psychiatric diagnosis, and psychopharmacological medication.

**Conclusions:**

Compared to the general population, we found substantially lower HRQOL in OST patients, especially in their mental HRQOL. OST programs can benefit from further improvement, particularly with regard to mental health services, in order to better serve their patients’ needs. Clinicians may consider the use of patient-reported outcome measures to identify patients’ subjective physical and psychological needs. Further research is needed to determine if employment is a cause or consequence of improved HRQOL.

**Trial registration:**

ClinicalTrials.gov: NCT02395198, retrospectively registered 16/03/2015

## Background

Opioid substitution treatment (OST) is an evidence-based intervention for opioid dependence that improves patients’ health and reduces the mortality rate [1–3]. The proportion of people who inject drugs (PWID) who receive OST varies greatly between countries. While coverage is estimated to be greater than 40 OST recipients per 100 PWID in western Europe and Australia, estimates for the USA, China, India, and Eastern Europe vary between 1 and 20 OST recipients per 100 PWID, and in most parts of the world OST is still not even available [4]. There are also large differences in sociodemographic and drug use characteristics in PWID worldwide. For example, PWID who are younger than age 25 make up less than 20% of PWID in North America, Australasia, Central Asia, and the Caribbean, but more than 40% of PWID in Eastern Europe and Latin America [5]. In Europe, the proportion of patients aged over 40 entering treatment for opioid use increased from 1 in 5 in 2006 to 1 in 3 in 2013 [6, 7]. This reflects an ageing cohort of opioid users who started injecting during the heroin “epidemics” of the 1980s and 1990s and who have shaped and characterized the current European treatment systems [6, 7]. A steadily increasing age of the OST population is also observed in many other regions in the world with a longer history of OST implementation, such as New York City [8] and Australia [9]. Thus, long-term OST patients are getting older and few young people are entering OST. An ageing population places increasing demands on the health care system [10, 11]. Especially the next two decades will pose a challenge, as the large cohorts of opioid users who initiated use in the eighties and nineties are growing old.

Understanding the needs of OST patients is critical to providing the right care. Health-related quality of life (HRQOL) is a valuable outcome measure in this regard. It is a concept that includes subjective physical and mental wellbeing, and can be useful in the evaluation of treatment programs and patient progress by providing insight from the patient’s perspective. [12].

Over the past two decades, there has been an increasing interest in the HRQOL of OST patients. Research consistently shows that OST patients’ HRQOL is significantly lower at treatment entry compared to the general population or people with psychiatric disorders [10, 13–15] and that HRQOL improves in the first months of OST [16–19]. However, comparatively little is known about the HRQOL of long-term OST patients. A few cross-sectional studies suggest that OST patients continue to have poor HRQOL in OST, but these studies are limited by small sample sizes and partially conflicting outcomes [10, 13, 20, 21]. Only Wittchen et al. (2011) assessed the HRQOL of a large sample of OST patients – although only as a secondary outcome – and found significantly lower HRQOL compared to the general population and no improvements over a one-year period [22].

In fact, while HRQOL improves at treatment uptake, the effect seems short-lived. Wang et al. (2012) conducted an 18-month study in which they assessed quality of life (QOL) every 3 months [23]. They found that QOL improved rapidly in the first 3 months of OST, but then the effect slowed down. Likewise, Ponizovsky et al. (2007) detected an improvement of QOL only in the first month of OST [24]. Habrat et al. (2002) demonstrated that while HRQOL improved significantly the first 6 months of OST, it then decreased again [25]. Karow et al. (2011) found that QOL increased more during the first 6 months of OST than the following 6 months and that it did not reach the level of healthy individuals [15]. Taken together, the literature suggests that OST is effective in enhancing QOL and HRQOL at treatment entry but may have shortcomings in the long-term.

Understanding the needs of subgroups of patients is essential to be able to provide appropriate care. Research consistently shows that female OST patients have poorer overall HRQOL than male patients [15, 21]. However, some studies found an association of gender with mental HRQOL, some with physical HRQOL, and others with both physical and mental HRQOL or neither [20, 26–29]. Similarly, there is conflicting evidence with regard to other factors, such as active drug use and hepatitis C virus (HCV) infection [13, 15, 30–32]. The present study aims to provide the first comprehensive data on the HRQOL of a large national sample of patients in OST including sociodemographic and clinical correlates.

## Methods

### Study design

This investigation is part of the larger study ‘Epidemiology Of Hepatitis C Virus Infection Among People Receiving Opioid Substitution Therapy (ECHO)’, an observational longitudinal multicentre study, which aims to estimate the national prevalence and incidence of HCV infection among OST patients in Germany. Stratified random sampling was performed to obtain a representative sample of OST clinicians based on their distribution according to German Federal State and the number of patients per clinician. For patients to be eligible to participate in the study they had to be diagnosed with opioid dependence according to the ICD-10, be currently in OST, be at least 18 years of age, and have sufficient German literacy skills. Patients were eligible to participate with any form of OST (e.g. liquid, pills, capsules) and any type of OST medication (e.g. methadone, buprenorphine, pharmaceutical heroin). OST physicians invited their patients to participate in the study; participation was voluntary and remuneration was provided. Once patients completed the questionnaire, they placed it in an envelope and sealed it so that physicians could not access the data. The study design is described in full detail elsewhere [33]. Ethical approval for the ECHO study was granted by the Ethics Committee of the Medical Association of Hamburg, Ref. PV4603, and by each local Ethics Committee in Germany.

### Study sample

From July 2014 to October 2016, epidemiological cross-sectional data was collected from a large national sample of 2474 outpatients receiving OST from 63 OST clinicians in Germany. A good regional distribution of clinicians across Germany was obtained, although smaller clinics were somewhat underrepresented in our sample. Of the 2474 patients, a total of 298 (12.1%) were excluded because they did not fill in the patient questionnaire (N=239, 9.7%) or because the HRQOL instrument had more than two missing values per participant (*N*=59, 2.4%; [34]). Of the final 2176 patients included in the analyses, clinician data was not available for 79 patients.

### Measures

HRQOL was measured with the 12-Item Short Form Health Survey (SF-12; [35–37]). The 12 items are a subset of the 36-item Short Form Health Survey (SF-36; [38]), and assess subjective functional health and wellbeing (e.g. “Does your health limit you in climbing several flights of stairs? If so, how much?” or “In the past week, how often were you calm and relaxed?”). Physical and Mental Component Summary Scores (PCS and MCS) are calculated. We chose the SF-12 because it is one of the most widely used HRQOL instruments in the addiction literature [39, 40] and because country-specific general population norms are available for it [36]. Good psychometric properties of PCS and MCS are reported for both the original American version and the German version used in this study (e.g. test-retest (2-week) correlations of 0.89 and 0.76 [37], and internal consistency (Cronbachs alpha) > .70 [36]). The SF-12 has a high construct validity in discriminating between patient groups known to differ in physical and mental conditions, and it is also sensitive to change [36, 37]. Correlations between the 12-item and the 36-item PCS and MCS are very high, ranging from 0.94–0.96 for PCS and 0.94–0.97 for MCS across different countries and languages [35].

Patients and clinicians completed questionnaires independently from one another.

Patients provided sociodemographic data (gender, age, employment, children, relationship, housing, and migration background) and completed the SF-12, the Brief Symptom Inventory-18 (BSI-18; [41]), and the Opiate Treatment Index Health Symptoms Scale (OTI-HSS; [42, 43]). The BSI-18 is a self-report measure of psychological distress, comprising a symptom checklist and yielding three sub-scores (Depression, Anxiety, and Somatization), as well as the Global Severity Index (GSI). The OTI-HSS is a self-report measure of physical health, comprising a checklist of 50 symptoms that opioid users often experience.

Clinicians provided clinical data (duration of current OST, substitution medication, years of opioid dependence, active drug use, HCV infection, human immunodeficiency virus (HIV) infection, psychiatric diagnosis, and psychopharmacological medication during the past 6 months) and rated patients’ functioning and illness severity using the Global Assessment of Functioning scale (GAF; [44]) and the Clinical Global Impression scale (CGI; [45]). Active drug use was defined as the consumption of at least one illegal substance (cocaine, benzodiazepines, heroin or amphetamine) once during the past three months. The last three urine samples from the past three months were tested for the four substances, thus creating 12 possible data sets per patient. Information on psychiatric diagnosis and psychopharmacological medication, as well as HCV and HIV status, was taken from the patients’ medical records. Clinicians were encouraged to perform HCV diagnoses in accordance with the German HCV testing-guidelines (i.e. yearly antibody tests for patients with negative serostatus). However, due to the non-interventional nature of our study, clinicians were not obliged to do this. The time of testing was therefore individual for each participant.

### Statistical Analysis

We calculated the two component summary scales of the SF-12 (PCS and MCS) in accordance with the German test manual [36], using US-derived item weights. Up to two missing values per participant were imputed in the SF-12 (method proposed by Perneger et al. [34]). Participants with more than 2 missing items were excluded from analysis. To compare the SF-12 scores of our sample with the general population, we calculated independent sample t-tests using PCS/MCS means, standard deviations and sample sizes of the German normative sample from 1998 [36]. In addition, we determined the percentages of patients scoring lower or higher than one standard deviation below or above the German general population mean. Bivariate associations between PCS/MCS and our variables of interest were assessed using Pearson’s correlations for continuous variables (e.g. age, BSI-18), independent samples t-tests for dichotomous variables (e.g. gender), and one-way ANOVAs for categorical outcomes (e.g. partnership, age groups). For each statistically significant association, we determined effect sizes (standardized mean difference (d) and partial eta^2^). In addition, we calculated multiple linear regression models to predict PCS and MCS based on sociodemographic and clinical characteristics. We included the variables gender, duration of opioid dependence, employment, living together with children, relationship, migration background, percentage of positive urine samples, duration of current OST, HIV status, and HCV status. The variables were selected based on considerations of relevance and multicollinearity. We first included all sociodemographic and clinical variables (Table 1) in one regression model (simultaneous entry), and then removed predictors that were either redundant in content or demonstrated intercorrelations higher than *r* = .6.

**Table 1:** Sociodemographic and clinical characteristics

Variables	Percentage or Mean (SD)
Male (*n* = 2176)	72.2 %
Age (*n* = 2176)	41.81 (8.94), range 18 - 70
18-30 years	9.8%
31-40 years	36.5%
41-50 years	35.4%
51-60 years	16.7%
61-70 years	1.6%
Employment (*n* = 2168)	
Full time/ education/ training	24.4%
Part time, regularly	10.1%
Occasional / Other	13.2%
Unemployed	52.3%
Having children (*n* = 1890)	52.4%
Living together with children (*n* = 2176)	19.7%
Relationship (*n* = 2158)	
Single	55.2%
Relationship, not living together	11.5%
Relationship, living together	33.3%
Own apartment (*n* = 2163)	83.1%
Migration background (*n* = 2166)	23.8%
Active drug use (past 12 weeks) (*n* = 1981)	36.1%
Percentage of positive urine samples (*n* = 1981)	10.04 (16.79), range 0 - 100
≥ 1 positive urine sample (past 4 weeks)	
Heroin (*n* = 1605)	16.3%
Cocaine (*n* = 1548)	5.3%
Benzodiazepines (*n* = 1598)	15.9%
Amphetamines (*n* = 1152)	5.3%
Years of opioid dependence (*n* = 2069)	20.37 (9.11), range 0 - 48
Duration of current OST (years) (*n* = 2048)	6.25 (5.21), range 0 - 26.5
Substitution medication (*n* = 2090)	
D-/L-Methadone	76.6% , mean dosage 97.0 (50.1) mg
Buprenorphine	22.6% , mean dosage 9.8 (6.2) mg
Other	0.8%
HCV status (*n* = 1929)	
Anti-HCV negative	43.6%
RNA negative / Anti-HCV positive	29.4%
RNA positive	26.9%
HIV positive (*n* = 1581)	3.7%
HIV/HCV co-infection (n = 1458)	2.2%

*Note*. Percentages are based on valid numbers, which are indicated in parentheses behind the variables

## Results

### Sample characteristics

Respondents (N = 2176) were predominantly male (72.2%) with a mean age of 41.8 (± 8.94) years. They were opioid dependent for an average of 20.4 (± 9.11) years and in their current OST for an average of 6.3 (±5.21) years. Of the total sample, 83.1% reported stable housing, 52.4% had children, 44.8 % were in a relationship, 34.5% were employed, and 23.8% had a migration background. Most respondents received methadone (76.6%), followed by buprenorphine (22.6%) and other substitution medications (0.8%). Moreover, 27.2% of participants were HCV-RNA positive, 3.7% were HIV-positive, and 2.2% were HCV/HIV co-infected. Thirty-six percent had consumed drugs within the past 12 weeks (Table 1).

To test for selection bias, we compared sample characteristics of the 2176 included patients with the 298 non-included patients, using data provided by the clinicians. Significant differences but with small effect sizes (around d = 0.3) emerged between the included and the non-included sample in (non-German) citizenship (10.0% vs. 18.9%), mean GAF ratings (65.7 ± 18.8 vs. 59.7 ± 19.1), mean CGI-S ratings (2.9 ± 1.6 vs. 3.5 ± 1.6), and past 4 weeks benzodiazepine use (15.7% vs. 23.9%). Only very small differences (d < 0.3) were found in age, gender, duration of current OST, and CGI-I ratings. No differences were found in psychiatric diagnosis, psychopharmacological medication, substitution medication, duration of opioid dependence, and use of heroin, cocaine or amphetamine.

### HRQOL of OST patients compared to the German normative sample

OST patients had a mean PCS of 44.63 (SD 9.75, range 11.04 - 64.08) and a mean MCS of 41.76 (SD 11.40, range 10.83 - 69.06; Table 2). Respondents scored significantly lower on the PCS than the German normative sample (M = 48.22, SD = 8.77; t(8850) = -15.270, *p*<0.001, d = -0.40). This effect was even more pronounced for the MCS (M = 51.41, SD = 8.55; t(8850) = -36.275, *p*<0.001, d = -1.03).

**Table 2:** Measures of HRQOL, physical health and mental health

Variables	Percentage or Mean (SD)
SF-12 PCS (*n* = 2176)	44.63 (9.75), range 11.04 - 64.08
SF-12 MCS (*n* = 2176)	41.76 (11.40), range 10.83 - 69.06
BSI-18 total, raw score (*n* = 2164)	13.33 (11.52), range 0 - 64
BSI-18 Somatization	3.58 (3.60), range 0 - 22
BSI-18 Depression	5.78 (5.28), range 0 - 24
BSI-18 Anxiety	3.97 (4.24), range 0 - 23
OTI-HSS sum score, gender-corrected (*n* = 2131)	11.99 (7.41), range 0 - 42.71
CGI-S (*n* = 2041)	2.91 (1.59), range 1-7
CGI-S categories (*n* = 2041)	
1 - Normal, not at all ill	27.5%
2 - Borderline mentally ill	16.9%
3 - Mildly ill	18.6%
4 - Moderately ill	17.6%
5 - Markedly ill	13.9%
6 - Severely ill	5.0%
7 - Extremely ill	0.5%
CGI-I (*n* = 1995)	2.71 (1.04), range 1 - 6
CGI-I categories (*n* = 1995)	
1 - Very much improved	10.3%
2 - Much improved	34.9%
3 - Minimally improved	33.4%
4 - No change	17.0%
5 - Minimally worse	3.1%
6 - Much worse	1.2%
GAF (*n* = 2076)	65.71 (18.81), range 0 - 100
Prevalence of psychiatric disorders ^a^(*n* = 2176)	56.9%
In psychopharmacological treatment (*n* = 2049)	27.5%

*Note.* SF-12, BSI-18, and OTI-HSS are patient-reported; CGI and GAF are clinician-reported; data on psychiatric disorders and psychopharmacological treatment is taken from patients’ medical records. Percentages are based on valid numbers, which are indicated in parentheses behind the variables. ^a^ in past 6 months: depression, anxiety disorder, PTSD, psychotic disorder, or Other,

*Abbreviations*. *SF-12* 12-Item Short Form Health Survey, *PCS* Physical Component Summary, *MCS* Mental Component Summary, *BSI-18* Brief Symptom Inventory-18, *OTI-HSS* Opiate Treatment Index - Health Symptoms Scale, *CGI-S* Clinical Global Impression – Severity, *CGI-I* Clinical Global Impression – Improvement, *GAF* Global Assessment of Functioning

**Fig. 1:**
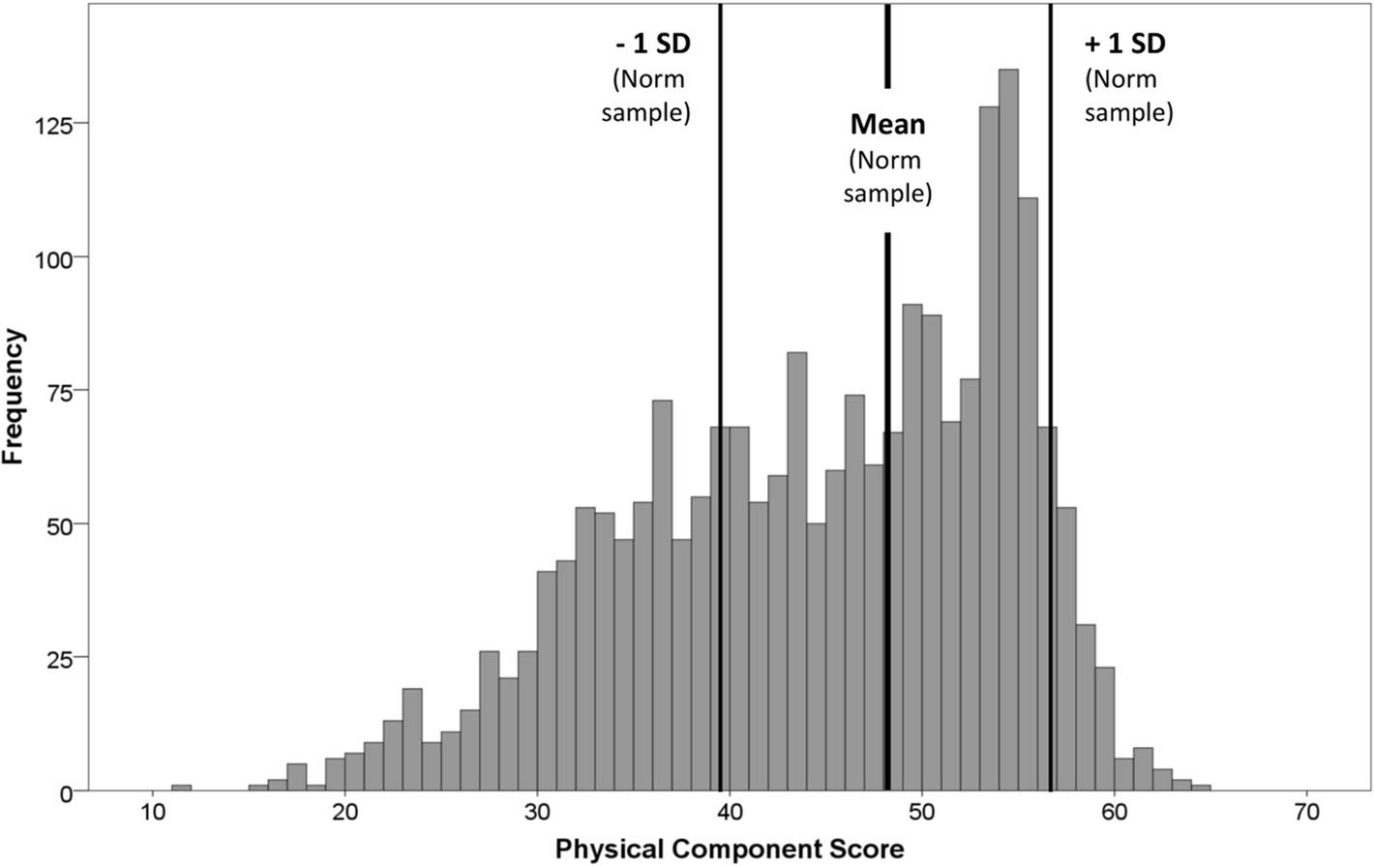
Distribution of the SF-12 Physical Component Summary score (PCS) compared with German general population norms. ECHO study sample (*n* = 2176) statistics: mean = 44.63, standard deviation = 9.75, range 11.04 – 64.08; skewness = -0.50, SE = 0.05; kurtosis = -0.56

**Fig. 2:**
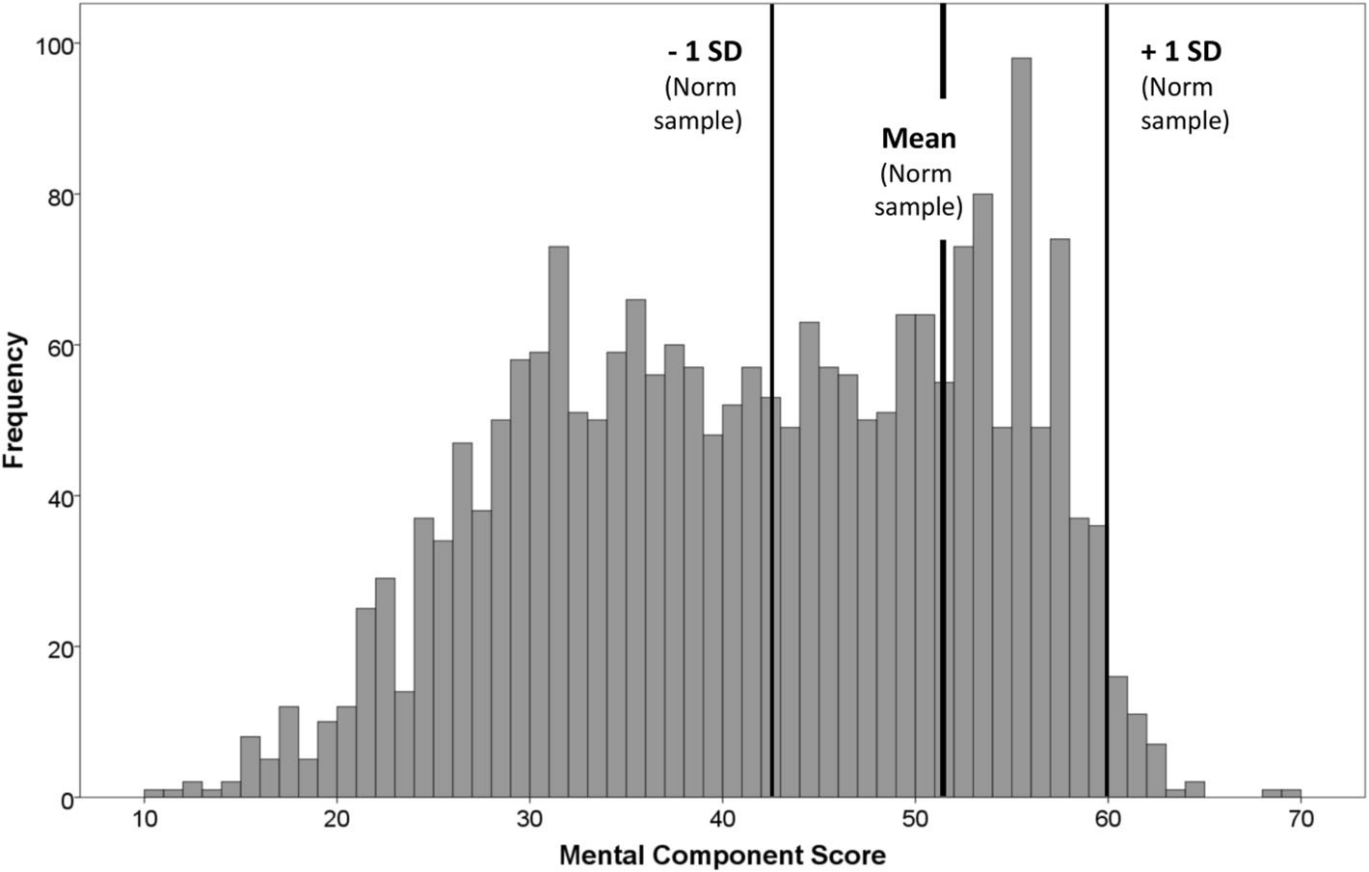
Distribution of the SF-12 Mental Component Summary score (MCS) compared with German general population norms. ECHO study sample (*n* = 2176) statistics: mean = 41.76, standard deviation = 11.40, range 10.83 – 69.06; skewness = -0.18, SE = 0.05; kurtosis = -0.95

The distributions of PCS and MCS scores in our sample are not bell-shaped. The PCS distribution is left-skewed, with a peak at about 55 points (Fig. 1). The MCS has a bimodal distribution with peaks at about 30 and 55 points (Fig. 2). For PCS, 30.4% of patients scored lower than one standard deviation (SD) below the German normative sample mean and 5.9% of patients scored higher than one SD above the mean (Fig. 1). For MCS, 51.7% of patients scored lower than one SD below the German normative sample mean and 1.8% of patients scored higher than one SD above the mean (Fig. 2). Regarding PCS and MCS together, 1362 patients (62.6% of the total sample) scored lower than one SD in at least one scale and, of this group, 425 patients (19.5% of the total sample) scored lower than one SD in both scales.

**Fig. 3:**
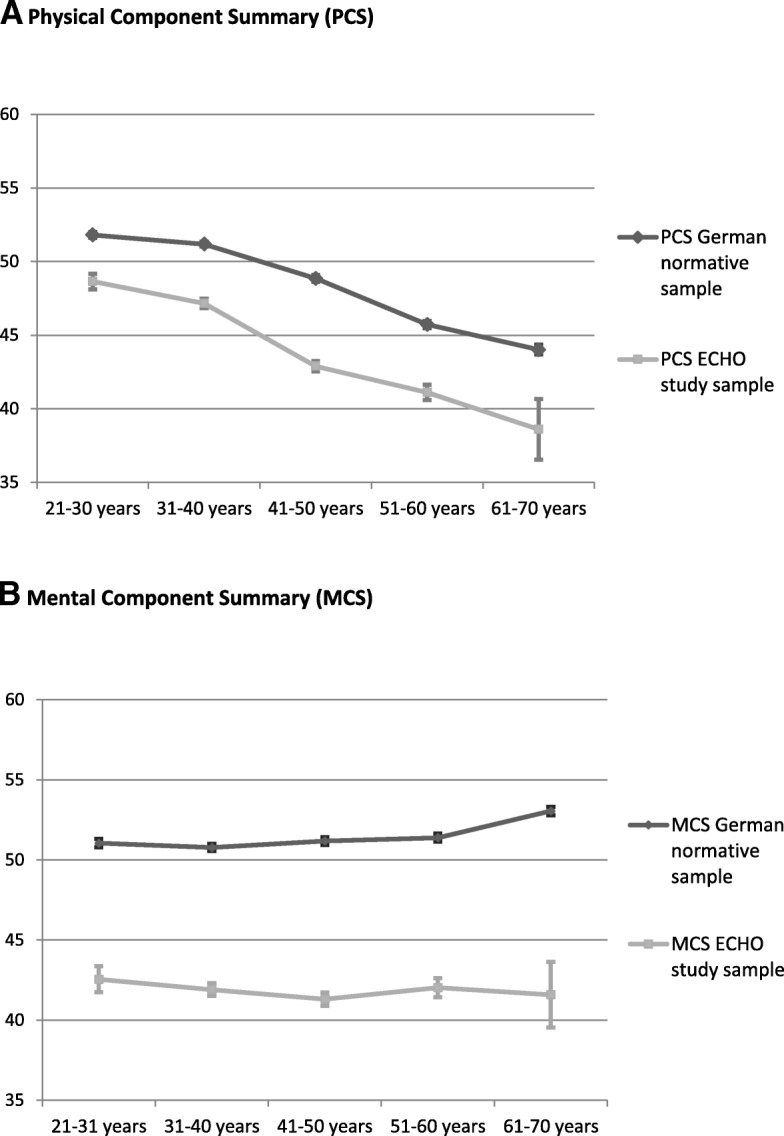
SF-12 scores by age groups, compared with the German general population. Means and standard errors of A: Physical Component Summary scores (PCS) and B: Mental Component Summary scores (MCS) by age groups for the study sample of OST patients (*n* = 2176) and the German normative sample (*n* = 6676)

Both male and female OST patients had lower SF-12 scores than the general population (Men PCS 44.58 ± 9.61 vs. 49.12 ± 8.20, d = -0.52; Men MCS 42.29 ± 11.13 vs. 52.54 ± 7.81, d = -1.14; Women PCS 44.79 ± 10.10 vs. 47.34 ± 9.19, d = -0.54; Women MCS 40.39 ± 11.98 vs. 50.30 ± 9.08, d = -1.07; all *p* <.001). Moreover, SF-12 scores were lower in all age groups compared to the general population (Fig. 3).

### Sociodemographic and clinical correlates of HRQOL in OST patients

Bivariate associations of sociodemographic and clinical variables with SF-12 scores are shown in Table 3. Relevant associations (effect sizes *d* > 0.35, *r* > 0.2, partial eta ^2^ > 0.04; Ferguson, 2009 [46]) are described first. Older age, longer duration of opioid dependence, methadone as substitution medication, HCV infection, and HIV infection were associated with lower PCS. Having a psychiatric diagnosis, being in psychopharmacological treatment, and current drug use were associated with lower MCS. Although, more specifically, only benzodiazepine use was associated with lower MCS, while amphetamine use was associated with higher PCS (no association with heroin or cocaine use). Unemployment was associated with lower PCS and MCS.

**Table 3:** Bivariate associations of sociodemographic and clinical variables with HRQOL in OST patients

		Physical Component Summary score (PCS)			Mental Component Summary score (MCS)		
		M (SD)	Statistics	Effect size	M (SD)	Statistics	Effect size
Gender	Male	44.58 (9.61)	*p* = .70	--	42.28 (11.13)	t(1027.85) = 3.31, *p* = .001	d = .16
	Female	44.77 (10.10)			40.42 (11.99)		
Age			*p* < .001	*r* = -.29		*p* = .50	--
Age	18-30 years	48.52 (7.73)	F (4, 2171) = 46.53, *p* < .001,	part. eta^2^ = .08	42.53 (11.75)	*p* = .62	--
	31-40 years	47.15 (8.76)			41.93 (11.21)		
	41-50 years	42.88 (9.89)			41.28 (11.52)		
	51-60 years	41.13 (10.04)			41.99 (11.34)		
	61-70 years	38.60 (12.06)			41.58 (11.98)		
Employment	Full time/education/training	49.56 (7.56)	F (3, 2164) = 109.84, *p* < .001	part. eta^2^ = .13	46.91 (10.34)	F (3, 2164) = 61.22, *p* < .001	part. eta^2^ = .08
	Part time, regularly	48.32 (8.43)			43.30 (11.13)		
	Occasional /other	45.32 (9.44)			41.26 (11.20)		
	Unemployed	41.50 (9.72)			39.21 (11.13)		
Having children	Yes	44.94 (9.69)	*p* = .47	--	42.10 (11.65)	*p* = .27	--
	No	44.61 (9.94)			41.51 (11.38)		
Living together with children	Yes	46.73 (9.43)	t (2174) = 5.00, *p* < .001	d = .27	44.70 (11.53)	t (2174) = 6.00, *p* < .001	d = .32
	No	44.12 (9.75)			41.04 (11.26)		
Relationship	Single	43.62 (9.90)	F (2, 2155) = 17.13, *p* < .001	part. eta^2^ = .02	40.67 (11.23)	F (2, 2155) = 20.33, *p* < .001	part. eta^2^ = .02
	Relationship, not living together	45.10 (9.41)			40.74 (10.87)		
	Relationship, living together	46.26 (9.28)			43.98 (11.58)		
Housing	Own apartment (rent)	44.87 (9.72)	t (2161) = 2.16, *p* = .031	d = .18	42.20 (11.42)	t (2161) = 4.12, *p* < .001	d = .24
	With friends, institutional, homeless	43.67 (9.76)			39.52 (11.15)		
Migration background	Yes	45.66 (9.26)	t (907.51) = 2.79, *p* = .005	d = .14	42.14 (11.17)	*p* = .38	--
	No	44.33 (9.87)			41.64 (11.48)		
Active drug use (past 12 weeks)	No	45.15 (9.94)	t (1569.77) = 2.25, *p* = .025	d = .10	43.29 (11.24)	t (1979)= 7.61 *p* < .001	d = .36
	Yes	44.15 (9.27)			39.29 (11.21)		
Heroin (past 4 weeks)	No	44.74 (9.72)	*p* = .82	--	42.05 (11.39)	*p* = .071	--
	Yes	44.59 (9.19)			40.67 (10.87)		
Cocaine (past 4 weeks)	No	44.68 (9.68)	*p* = .84	--	41.88 (11.29)	*p* = .08	--
	Yes	44.90 (8.63)			39.66 (11.49)		
Benzodiazepines (past 4 weeks)	No	45.06 (9.67)	t (1596) = -3.76, *p* < .001	d = .26	42.80 (11.14)	t (1596) = -8.11, *p* < .001	d = .56
	Yes	42.58 (9.40)			36.64 (10.84)		
Amphetamines (past 4 weeks)	No	44.95 (9.50)	t (71.90) = 3.83, *p* < .001	d = .40	42.24 (11.07)	*p* = .07	--
	Yes	48.69 (7.29)			39.60 (11.52)		
Years of opioid dependence			*p* < .001	*r* = -.28		*p* = .30	--
Duration of current OST (years)			*p* < .001	*r* = -.08		*p* = .18	--
Substitution medication	D-/L- Methadone	43.88 (9.77)	t (842.3) = -7.89, p < .001	d = .39	41.06 (11.26)	t (2071) = -5.50, *p* < .001	d = .29
	Buprenorphine	47.62 (8.84)			44.31 (11.48)		
HCV-status	Anti-HCV -	47.13 (9.11)	F (2, 1926) = 48.68, p < .001	part. eta^2^ = .05	42.28 (11.72)	F (2, 1926) = 7.93, *p* < .001	part. eta^2^ = .01
	RNA - / Anti-HCV +	43.49 (9.46)			42.26 (11.03)		
	RNA +	42.31 (10.09)			39.96 (10.97)		
HIV-status	Positive	40.44 (10.20)	t (2174) = -3.36, p = .001	d = .44	39.02 (10.94)	*p* = .061	--
	Negative/unclear	44.75 (9.71)			41.84 (11.41)		
HCV/HIV-status (if both known)	HCV -/ HIV -	45.61 (9.50)	F (3, 1466) = 14.40, p < .001	part. eta^2^ = .03	41.84 (11.60)	F (3, 1466) = 3.01, *p* = .029	part. eta^2^ = .01
	HCV +/ HIV -	42.35 (10.00)			40.16 (11.03)		
	HCV -/ HIV +	40.31 (9.22)			40.48 (10.62)		
	HCV +/ HIV +	39.90 (11.03)			37.78 (11.62)		
BSI-18 total score			*p* < .001	*r* = -.41		*p* < .001	*r* = -.67
BSI-18 Somatization			*p* < .001	*r* = -.52		*p* < .001	*r* = -.45
BSI-18 Depression			*p* < .001	*r* = -.31		*p* < .001	*r* = -.70
BSI-18 Anxiety			*p* < .001	*r* = -.28		*p* < .001	*r* = -.56
OTI-HSS gender-corrected sum score			*p* < .001	*r* = -.48		*p* < .001	*r* = -.50
CGI-S (severity)			*p* < .001	*r* = -.23		*p* < .001	*r* = -.40
CGI-I (improvement)			*p* < .001	*r* = -.12		*p* < .001	*r* = -.17
GAF			*p* < .001	*r* = .35		*p* < .001	*r* = .37
Psychiatric diagnoses	1 or more	43.23 (9.85)	t (2174) = -7.83, *p* < .001	d = .34	38.63 (11.07)	t (2174) = -15.54, *p* < .001	d = .67
	None	46.49 (9.29)			45.90 (10.45)		
Psychopharmacological treatment	Yes	42.44 (9.85)	t (2047) = -6.56, *p* < .001	d = .33	36.89 (10.97)	t (2047) = -12.55, *p* < .001	d = .62
	No	45.56 (9.49)			43.70 (10.98)		

*Note*. Effect sizes and test statistics are reported for all *p* < .05

Abbreviations. *BSI-18* Brief Symptom Inventory-18, *OTI-HSS* Opiate Treatment Index - Health Symptoms Scale, *CGI-S* Clinical Global Impression – Severity, *CGI-I* Clinical Global Impression – Improvement, *GAF* Global Assessment of Functioning, *HCV* Hepatitis C virus, *HIV* Human immunodeficiency virus

Other significant associations but with very small effect sizes emerged (*d* < 0.35, *r* < 0.2, partial eta ^2^ < 0.04). Women exhibited slightly lower MCS than men, patients receiving methadone had lower MCS than those receiving buprenorphine, and patients with HCV infection had slightly lower MCS than those without. Psychiatric diagnosis, psychopharmacological treatment, longer duration of current OST and migration background was weakly associated with lower PCS. There was also a very small association between drug use and lower PCS. More specifically, only amphetamine use and benzodiazepine use was associated with lower PCS (and not the other two substances we measured, heroin and cocaine). Being in a relationship and living in stable housing was associated with slightly higher PCS and MCS. Participants who lived together with their children had slightly higher PCS and MCS than those who did not.

In the multivariate models (*n* = 1703; Table 4), higher PCS was predicted by (highest regression weights mentioned first) stable employment, shorter duration of opioid dependence, negative HCV status, the absence of a psychiatric diagnosis, and being substituted with buprenorphine. Higher MCS was predicted by (highest regression weights mentioned first) the absence of a psychiatric diagnosis, stable employment, not being in psychopharmacological treatment, less drug use, male gender, being substituted with buprenorphine and living together with children. As 473 patients were excluded from the multivariate model due to missing values, we checked for possible selection bias. The 1703 patients included in the regression model were slightly younger (41.5 years (± 8.8) vs. 42.9 (± 9.3) years, *p* = .004), reported less physical impairments (better PCS and BSI somatization subscale), were slightly longer in their current OST, and had higher CGI-I ratings (all differences d < .2). No differences in other variables emerged.

**Table 4:** Multivariate linear regression models of sociodemographic and clinical variables with HRQOL in OST patients (*n* = 1703)

	Physical Composite Score (PCS) Adjusted R^2^ = .206			Mental Composite Score (MCS) Adjusted R^2^ = .181		
	B (95% CI)	Beta	*p*	B (95% CI)	Beta	*p*
Gender	.00 (-.95 – .96)	.00	.99	**-1.91 (-3.04 – -.77)**	**-.08**	**.001**
Duration of opioid dependence (years)	**-.18 (-.24 – -.13)**	**-.17**	**<.001**	.05 (-.02 – .11)	.04	.13
Substitution medication	**1.75 (.73 – 2.77)**	**.08**	**.001**	**1.60 (.38 – 2.82)**	**.06**	**.010**
Employment	**-2.11 (-2.47 – -1.75)**	**-.28**	**<.001**	**-1.56 (-1.99 – -1.13)**	**-.17**	**<.001**
Living together with children	-.50 (-1.63 – .62)	-.02	.38	**-1.39 (-2.74 – -.05)**	**-.05**	**.042**
Relationship	.19 (-.31 – .68)	.02	.38	.13 (-.46 – .72)	.01	.66
Migration background	.34 (-.67 – 1.34)	.02	.51	1.17 (-.02 – 2.37)	.04	.06
Percentage of positive urine samples	-.01 (-.02 – .04)	-.02	.46	-.05 (-.08 – -.02)	-.08	<.001
Time in current OST	-.00 (-.01 – .00)	-.02	.34	.00 (-.00 – .01)	.02	.33
HIV status	2.1 (-.38 – 4.53)	.04	.10	1.38 (-1.56 – 4.31)	.02	.36
HCV status	**-1.0 (-1.56 – -.48)**	**-.09**	**<.001**	-.42 (-1.06 – .23)	-.03	.20
Psychiatric diagnoses	**1.68 (.72 – 2.65)**	**.09**	**.001**	**4.73 (3.58 – 5.89)**	**.21**	**<.001**
Psychopharmacological treatment	.85 (-.20 – 1.89)	.04	.11	**3.12 (1.87 – 4.37)**	**.12**	**<.001**

*Note.* Significant predictors are highlighted in bold. Variable codings and ranges are listed here. Gender: 1 = Male, 2 = Female; Duration of opioid dependence (years), range: 0 – 48; Substitution medication: 1 = D-/L-Methadone, 2 = Buprenorphine; Employment: 1 = Full time/education/training, 2 = Part time, 3 = Occasional /other, 4 = Unemployed; Living together with children: 1 = Yes, 2 = No; Partnership: 1 = Single, 2 = Relationship, not living together, 3 = Relationship, living together; Migration background: 1 = Yes, 2 = No; Percentage of positive urine samples, range: 0 – 100; Duration of current OST (months), range: 0 – 318; HIV status: 1 = Positive, 2 = Negative/unclear; HCV status: 1 = Anti-HCV - , 2 = Anti-HCV +/ RNA - , 3 = Anti-HCV +/ RNA + ; Psychiatric diagnoses: 1 = One or more, 2 = None; Psychopharmacological treatment: 1 = Yes, 2 = No.

### HRQOL and measures of physical and mental health

Better self-reported mental health (BSI-18) correlated moderately with better PCS (*r* = -.41) and strongly with better MCS (*r* = -.67). More specifically, the BSI-18 subscale Somatization correlated moderately with PCS and MCS, while the subscales Depression and Anxiety correlated weakly with PCS and strongly with MCS. The OTI-HSS score correlated moderately with PCS (*r* = -.48) and MCS (*r* = -.50), such that participants with a higher OTI-HSS score demonstrated lower PCS and MCS (Table 3). Clinicians’ ratings of patients’ mental illness severity (CGI-S: M=2.91, SD=1.59, range 1-7) and functioning (GAF: M=65.72, SD=18.81, range 0-100) were weakly correlated with patients’ self-reported physical and mental HRQOL (Table 2 and 3).

## Discussion

This study presents a comprehensive and differentiated assessment of the physical and mental HRQOL of a large national sample of OST patients in Germany. Substantial impairments were found in OST patients’ HRQOL, especially in their mental HRQOL. However, there was also a smaller subgroup of patients with considerably better HRQOL than the rest of the sample, indicating that it is possible for patients to attain a relatively good HRQOL. This suggests that there is room for improvement in OST programs, particularly relating to patients’ mental wellbeing.

Our findings may inform tailored interventions for subgroups of patients and have implications for drug policies. For example, age was one of the most important correlates of poor physical HRQOL, suggesting that older OST patients may benefit from enhanced health care services. Moreover, the association of chronic HCV infection with low physical HRQOL highlights the importance of providing antiviral HCV treatment to OST patients. Many clinicians still hesitate to provide HCV treatment to drug users, because they fear reinfection or non-adherence to treatment, and also drug-users are frequently unwilling to take up HCV treatment [47]. However, especially in this new era of direct-acting antiviral (DAA) treatment with reduced side effects and high rates of sustained virologic response (SVR), it is important to reduce the barriers to HCV diagnosis and treatment and to educate clinicians and patients about treatment benefits beyond SVR, such as increased subjective wellbeing and reduced symptoms of extra-hepatic manifestations [48, 49]. While it makes sense that older age, HCV and HIV infection are associated with lower physical HRQOL, it should be noted that these factors have a greater impact on drug users than non-drug users [50, 51], highlighting the need for additional support specifically for older drug users.

While OST briefly improves mental health outcomes at the beginning of treatment [52], it does not appear to address patients’ mental health adequately in the long run. Opioid dependent individuals have high levels of psychiatric symptoms [22, 53]. Past-year prevalence estimates of co-occurring psychiatric disorders range between 30% and 50% for mood disorders (e.g. depression) and 10% to 20% for anxiety disorders [54, 55]. However, our study and a recent 6-year follow up cohort study demonstrate that this high psychiatric comorbidity persists in long-term OST patients [56]. More than half of the patients in our sample had at least one psychiatric diagnosis. This is an important finding, because patients with dual diagnosis face barriers to adequate mental health treatment, including insufficient cooperation between mental and medical health institutions and the under-identification of dual diagnosis, which is in part due to the lack of mental health training in physicians [57–59]. As our study finds that psychiatric diagnosis and psychopharmacological treatment are associated with both mental and physical HRQOL, one may argue that it is particularly important to address OST patients’ mental health, as it is not only associated with mental but also physical wellbeing.

Gender was only weakly associated with HRQOL. This may explain the mixed findings in the literature from smaller studies, which show associations of gender with mental or physical HRQOL or neither or both [15, 20, 21, 26–29]. Similarly, there were mixed findings in the literature on the association of HRQOL with active drug use. Our results show that only benzodiazepine and amphetamine use was associated with HRQOL and not heroin or cocaine use. Benzodiazepine users might be self-medicating, considering that they often have a more complicated course of OST and exhibit more poly drug use [60]. Moreover, the regular and long-term use of benzodiazepines itself reduces quality of life and has adverse effects like cognitive or psychomotor impairment [61, 62].

While we found an association of HRQOL with substitution medication, the differences between methadone and buprenorphine are likely confounded with other factors that correlate with buprenorphine prescription, such as age, duration of opioid dependence, and preexisting physical and mental health. Buprenorphine is less sedating than methadone, but it is also more often prescribed to younger and more stable patients. The complexity of these interrelations needs to be considered when interpreting the results, and is also reflected in the multivariate model where the association between OST medication and HRQOL becomes much smaller when controlling for the above-mentioned factors.

In the multivariate model, we only included sociodemographic and clinical predictors, because bivariate associations of PCS and MCS with self-reported physical and mental health were already demonstrated. Consequently, the percentage of variance explained was relatively low (21% for PCS, 18% for MCS), which is however not surprising, given that HRQOL is influenced by a range of factors that cannot all be measured in a study. With regard to a potential selection bias that may have resulted from the listwise inclusion in our multivariate analyses, we consider the subsample included in the regression models highly representative for the total sample; differences in age and health are only marginal and no other relevant differences emerged. Results of bivariate and multivariate analyses are highly comparable. The most important bivariate correlations were also found as predictors in the multivariate model, and the relative importance of each predictor (expressed by standardized beta weights) reflects the effect sizes determined via bivariate comparisons. The most important predictors for both PCS and MCS were employment and mental health, followed by duration of opioid dependence for PCS.

Another interesting observation is that, even though patients and clinicians provided ratings for HRQOL and functioning independently, the scores correlated. Patients’ self-reported mental and physical HRQOL correlated with clinician-rated patient functioning (GAF) and clinician-rated mental illness severity (CGI-S) (Table 3). The literature often reports a discrepancy between the perspectives of patients and clinicians [63, 64], but our findings suggest that in patients with an opioid use disorder, clinicians’ ratings of functioning and mental illness severity are good indicators of patients’ HRQOL.

HRQOL correlated with patient-reported measures of physical and mental health. These “cross-over” associations between the physical and mental domains (i.e. MCS with OTI-HSS, and PCS with BSI-18) suggest that there is not a strict division between physical and mental health with regard to their impact on a person’s subjective wellbeing. Nevertheless, brief symptom-based psychiatric screening tools should be implemented more regularly in clinical practice, given the high prevalence of mood and anxiety disorders in the opioid dependent population. Systematic screening for depression and anxiety, including in newly admitted patients, can reduce under-identification of comorbid disorders, which is a structural barrier to mental health treatment [58, 65]. Practitioners should use instruments with good validity for drug using populations, such as the BSI-18 [66].

As our study had a cross-sectional design, we could not determine if unemployment was a cause or consequence of poor HRQOL. Considering unemployment status was the most important factor correlating with HRQOL, future research should investigate this relationship further. Moreover, longitudinal studies could evaluate the effects of work rehabilitation programs on OST patients’ HRQOL, and qualitative studies could investigate patients’ perspectives and needs with regard to employment and HRQOL. It is also important to clarify if and how re-integration in the labor market is a reasonable treatment goal for opioid dependent patients. So far, in Germany, work rehabilitation plays virtually no role in OST in practice [67].

A limitation of this study is its possible selection bias. Due to missing or incomplete patient questionnaires, 358 patients (14.5%) had to be excluded. Differences between the included and excluded samples were small, although it is worth noting that excluded patients had greater impairments in (clinician-reported) mental health and functioning, meaning the HRQOL of this study sample might be higher than that of the actual overall population of OST patients. A second limitation is the interpretation and validity of our drug use and treatment variables. As we collected routine data, which differed between study sites, the frequency of urine sampling and the substances that were tested varied between OST practices. Moreover, we did not record which patients were prescribed benzodiazepine, so that we cannot distinguish between prescribed and non-prescribed benzodiazepine use. We also did not keep track of what other interventions or services the participants were using and suggest that future research explores their additional impact on HRQOL. A third limitation is that we used data from the German normative sample from 1998. However, (a) the HRQOL of the German general population has improved since 1998, especially in individuals over the age of 50 [68], and (b) our sample is about 4 years younger and includes more men than the German general population and SF-12 norm samples [69, 70]. This is important, because younger age and male gender are associated with better HRQOL [68]. Therefore, if we compared our sample of OST patients to a more recent norm sample with younger and more male individuals, there would be an even bigger difference in HRQOL scores.

This study highlights the need for more patient-centered care. Rather than just focusing on clinical symptoms, we should also measure the subjective experiences and needs of OST patients to be able to provide more effective and patient-oriented interventions and care. HRQOL is a useful patient-reported outcome measure in this regard. Given the high comorbidity of opioid dependence and given that diseases and symptoms are burdensome to different degrees to different people, a measure of subjective wellbeing is arguably a better indicator of patients’ needs than symptom-based instruments such as the BSI-18 and OTI-HSS. It should be noted that the SF-12 is a generic HRQOL instrument and may therefore not provide sensitive data on the HRQOL of OST patients. Future research should use a drug-user specific HRQOL instrument with items that are relevant and specific to OST patients.

The differences in mental and physical HRQOL of OST patients demonstrate the need to measure wellbeing in multiple life domains. However, also the concept of HRQOL is limited in its scope and future research should examine the broader concept of QOL for a more comprehensive understanding of patients’ wellbeing and a holistic approach to patient’s recovery. The concept of QOL goes beyond symptoms of physical and mental health and broadens the view on a person’s condition by including aspects such as social and economic participation. Given that opioid dependence is a complex chronic disease, the improvement of QOL is a more adequate treatment goal than the absence of symptoms. To monitor long-term treatment success, a short but reliable QOL instrument, such as the Opioid Substitution Treatment Quality of Life scale (OSTQOL, [71]) could be a useful tool for OST providers.

## Conclusions

Compared to general population norms, we found substantially lower HRQOL in OST patients, especially in their mental HRQOL. Interestingly, our sample also comprised a considerable albeit smaller proportion of high-functioning OST patients with good physical and mental health, employment, stable housing, and/or stable family situation. However, the biggest proportion of OST patients had severe deficits in physical and mental health and HRQOL, suggesting that OST programs could benefit from further improvement to better serve their patients’ needs, particularly with regard to their mental health. An integrated health care approach is needed in which different physical and mental health care services are offered in combination, such as psychosocial support, therapy, and case management, as well as medical care specializing in the physical problems of opioid users. Moreover, more patient-centered care is needed to incorporate the patients’ perspectives and experiences in the treatment plan. Clinicians may consider the use of patient-reported outcome measures to enhance patient engagement in treatment.

## Abbreviations

BSI-18: Brief Symptom Inventory-18
CGI: Clinical Global Impression
ECHO: Epidemiology Of Hepatitis C Virus Infection Among People Receiving Opioid Substitution Therapy
GAF: Global Assessment of Functioning
GSI: Global Severity Index
HCV: Hepatitis C Virus
HIV: Human Immunodeficiency Virus
HRQOL: Health-Related Quality Of Life
MCS: Mental Component Summary (composite of the 12-Item Short Form Health Survey)
OST: Opioid Substitution Treatment
OTI-HSS: Opiate Treatment Index - Health Symptoms Scale
PCS: Physical Component Summary (composite of the 12-Item Short Form Health Survey)
QOL: Quality Of Life
SF-12: 12-Item Short Form Health Survey
SVR: Sustained Virologic Response
